# Supplementary figures and images for: Glucose Signaling Is Important for Nutrient Adaptation during Differentiation of Pleomorphic African Trypanosomes
Source: mSphere. 2018 Oct 31;3(5):e00366-18. doi: 10.1128/mSphere.00366-18 (PMC6211221; doi:10.1128/mSphere.00366-18)

A.

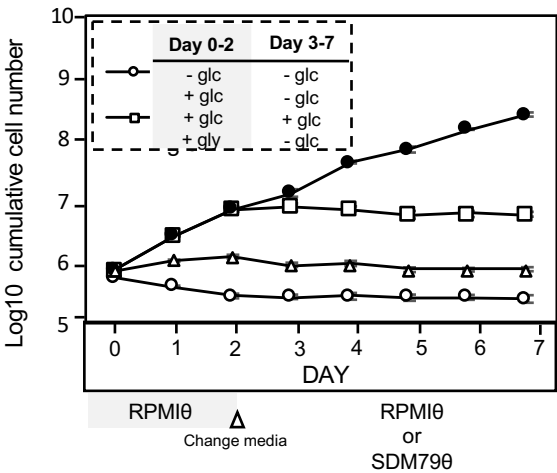

B.

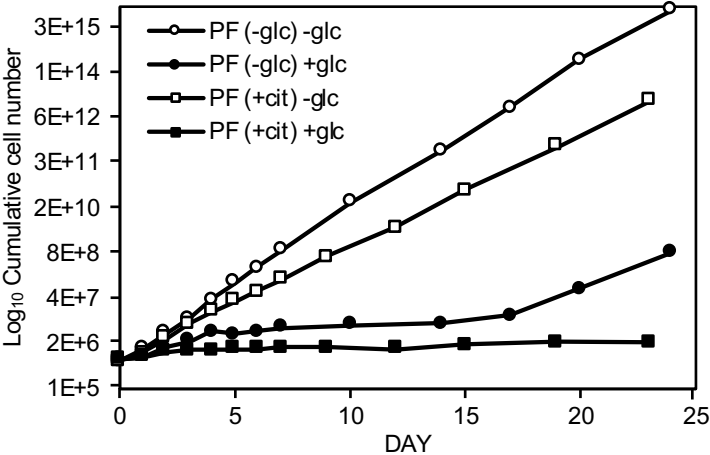

Supplement: FIG S1 [file sph006182679sf1.pdf]

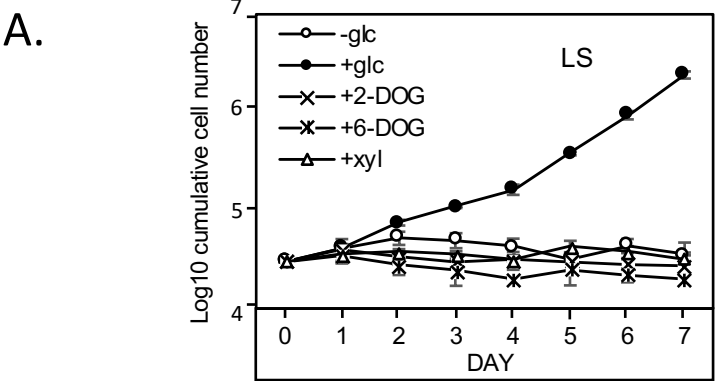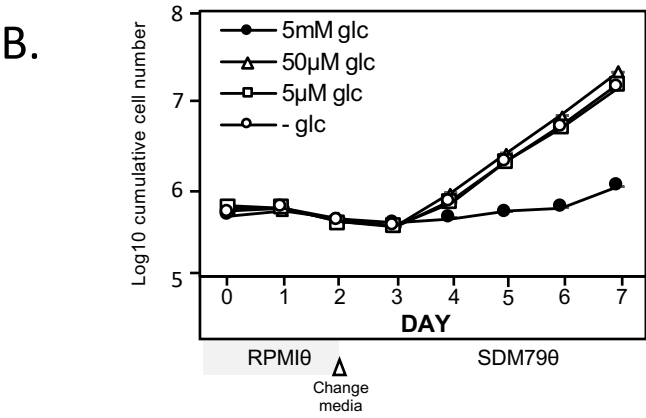

Supplement: FIG S2 [file sph006182679sf2.pdf]

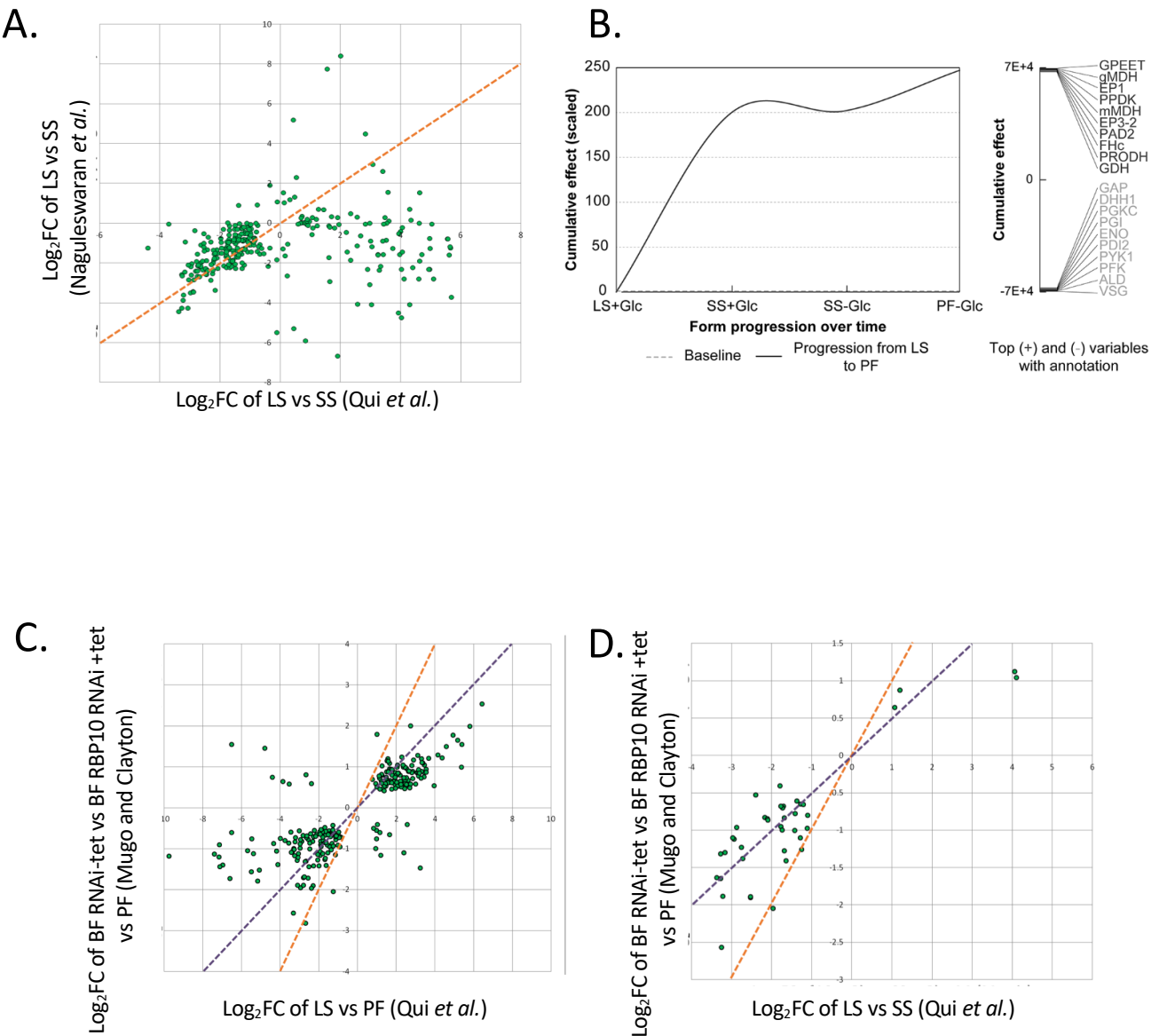

Supplement: FIG S3 [file sph006182679sf3.pdf]

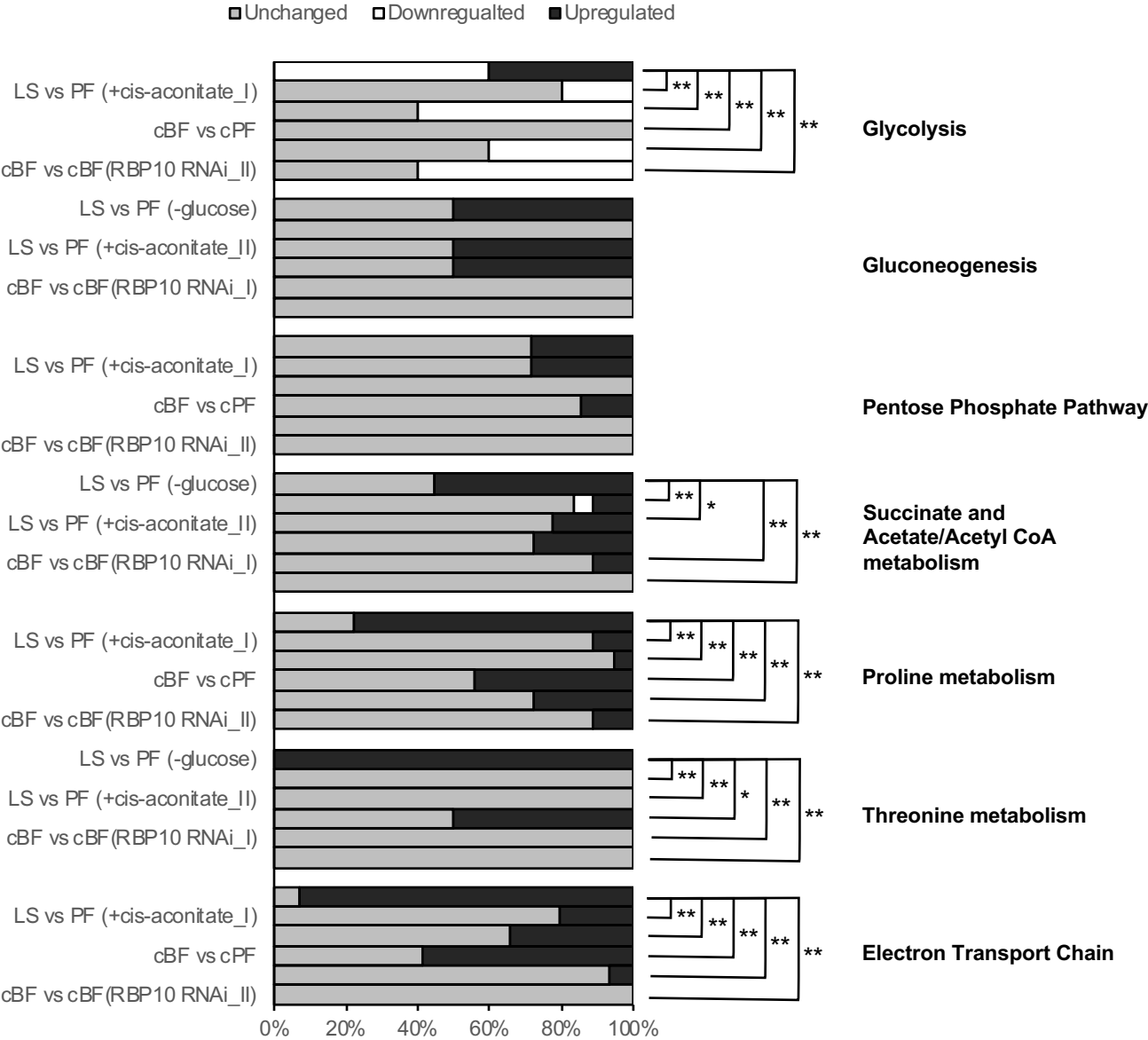

Supplement: FIG S4 [file sph006182679sf4.pdf]
